# Physiological Responses and Pregnancy Rate of Black and White Holstein Heifers During Hot and Cold Seasons in a Desert Region

**DOI:** 10.3390/ani15233458

**Published:** 2025-11-30

**Authors:** Leonel Avendaño-Reyes, Emiliano Corrales-Navarro, Ulises Macías-Cruz, María de los Ángeles López-Baca, José A. Roque-Jiménez, Peter H. Robinson, Miguel Mellado, Joaquín Hernández

**Affiliations:** 1 Instituto de Ciencias Agrícolas, Universidad Autónoma de Baja California, Valle de Mexicali 21705, Baja California, Mexico; lar62@uabc.edu.mx (L.A.-R.); emiliano.corrales@uabc.edu.mx (E.C.-N.); maria.lopez.baca@uabc.edu.mx (M.d.l.Á.L.-B.); jose.roque@uabc.edu.mx (J.A.R.-J.); 2 Instituto de Investigación en Ciencias Veterinarias, Universidad Autónoma de Baja California, Mexicali 21386, Baja California, Mexico; 3 Animal and Dairy Sciences Department, University of California, Davis, CA 48900, USA; phrobinson@ucdavis.edu; 4 Departamento de Nutrición Animal, Universidad Autónoma Agraria Antonio Narro, Saltillo 25315, Coahuila, Mexico; mmellbosq@yahoo.com; 5 Departamento de Patología Animal, Universidad de Santiago de Compostela, Campus Terra-IBADER, 27002 Santiago de Compostela, Spain; joaquin.hernandez@usc.es

**Keywords:** thermoregulation, Holstein heifers, blood biochemistry, infrared thermography, pregnancy rate

## Abstract

Our aim was to compare certain physiological traits and pregnancy rates in dairy heifers classified by coat color during summer and winter months in a Sonoran Desert region. Sixty Holstein heifers were randomly selected (age and weight of 13.1 ± 0.82 months and 382 ± 5.3 kg) and divided by season (i.e., winter and summer) and hair coat color (i.e., black and white). There were 28 summer heifers (13 black/15 white) and 32 in winter (18 black/14 white). Respiratory frequency (RF) was determined visually, and body surface temperatures (BSTs) of several anatomic regions were collected using thermography at 06:00 and 15:00 h every 3rd day. Furthermore, three blood samples were collected from each heifer in each season to determine hematological profiles and serum analytes (i.e., electrolytes, metabolites, hormones). Reproductive variables were pregnancy rate and services per pregnancy. Ambient temperatures ranged from 25 to 49 °C in summer and 3 to 28 °C in winter. In summer and winter, RF and most BST were higher (*p* < 0.05) in black heifers in the morning, while only head, right flank, and loin were higher (*p* < 0.05) in the afternoon. Cortisol and progesterone concentrations were higher (*p* < 0.01) in summer, while thyroid hormones were higher (*p* < 0.05) in winter. Glucose, triglycerides, cholesterol, urea, and sodium were higher (*p* < 0.05) in winter. Reproductive traits were unaffected by season or coat color. Although there were some differences in physiological variables and some biochemical parameters by season and coat color, these alterations were not enough to modify reproductive responses of Holstein heifers.

## Simple Summary

Global climate change is altering the biological responses of farm animals including dairy cattle raised outdoors in desert areas. This study examined how black and white Holstein dairy heifers responded to extreme summer and winter weather in a Sonoran Desert region, focusing on their physiological responses and the probability of pregnancy. Sixty heifers were grouped by coat color and season, and the physiological variables breathing rate, body surface temperature, and blood indicators related to stress, metabolism, and hormones were measured and evaluated. As expected, black-coated heifers tended to be hotter, especially during summer mornings. Stress and reproductive hormones also differed by season, but these differences failed to affect the pregnancy rate. Overall, coat color and season influenced some body functions but did not impact the reproductive performance of the heifers.

## 1. Introduction

Global climate change, defined as alterations in global meteorological parameters, especially the increase in ambient temperature, is an important alteration, particularly in already hot ecosystems. Indeed, recent high ambient temperatures in several temperate countries [1,2] often exceed the upper critical range for dairy cattle, leading to extended heat stress (HS) before and after the traditional summer period [3]. In this case, HS is defined as the sum of external stressors acting on the animal to trigger an increase in body temperature and other physiological responses [4]. From an environmental perspective, factors involved in the heat gain are ambient temperature, relative humidity, air velocity, and solar radiation. However, several natural processes, such as mastication, rumination, digestion, and fermentation, exacerbate the heat load [5], causing heat-stressed animals to activate a series of thermoregulation mechanisms (i.e., respiratory frequency, heart rate, and sweating rate) that benefit the homoeothermic response to the thermal challenge [6,7,8]. Indeed, HS is widely considered the main source of economic loss and welfare concerns for dairy cattle husbandry in the 21st century [9]. Overall, the current and expected incidence of extreme ambient heat is a critical challenge for dairy producers.

In dairy cattle production, heifers are the next generation of cowherds with genetic potential for future herd performance. Thus, efforts to develop a healthy female with high milk production and reproductive potential is critical. However, during growth and development, heifers are subjected to environmental factors that may delay conversion to maturity [10]. These factors include diet, facilities, climate, and management practices [11]. In hot and dry areas, environmental climatic stressors are main issues facing performance of dairy calves and heifers [9,10]. Heifers are considered more heat-tolerant than lactating cows because they generate less metabolic heat and have higher body surface area relative to body mass, and these differences facilitate body heat dissipation [6]. However, HS in heifers has a negative influence on rumen motility, feed efficiency, weight gain, hormone synthesis, duration and intensity of estrus, as well as ovarian follicular and embryo development [4,12]. There is also evidence that cooling pregnant dairy heifers causes a positive thermoregulatory response with changes that lead to improvements in production postpartum [13].

The association between hair coat color, thermoregulation, and growth traits in cattle has been of interest because hair color is considered a characteristic that impacts adaptation to hot climates [14,15]. A direct interaction between hair coat color and adaptation to ambient heat load explains how some cattle breeds acclimate to a particular ambient heat load [16]. The color of the hair coat in cattle may also affect body temperature and other physiological variables in cattle exposed to direct solar radiation in arid ecosystems. In hot desert regions [17], as well as in tropical regions [15], Holstein cows with a higher proportion of black color had higher body temperature and respiration frequency versus whiter cows. It is worth mentioning that the effect of coat color on the reproductive variables of heat-stressed Holstein heifers has not been extensively explored. However, the results in this regard are limited and generally agree in reporting no effect on pregnancy rate in multiparous cows [18,19]. Efforts need to be focused on determining the impact of coat color on physiological thermoregulation mechanisms and fertility.

Understanding how dairy heifers respond to HS by studying physiological and reproductive traits and identifying adaptive responses that Holstein heifers use to mitigate the impact of HS is important for simplifying the evaluation of cost-effective strategies for HS resilience. Because there is little evidence of how seasonal HS affects physiological and reproductive performance of Holstein heifers in hot–dry regions relative to hair coat color, we hypothesized that seasonal HS would cause physiological and biochemical alterations depending on coat color, thereby affecting pregnancy outcomes in heifers. Thus, the objective was to evaluate effects of seasonal heat stress and hair coat color on respiration frequency, body surface temperature, blood analyte concentrations, and pregnancy rate of Holstein heifers in a hot–dry region.

## 2. Materials and Methods

All heifer management practices followed accepted Mexican Official Standards NOM-051-ZOO-1995 (Human treatment during animal’s mobilization) and NOM-066-ZOO-1999 (Zoosanitary requirements for food products of animal origin). In addition, all experimental procedures were reviewed and authorized by the UABC Ethics Committee (Letter number: 058/2020-2).

### 2.1. Location and Duration of the Study

The study was completed on a heifer farm located in the Mexicali Valley, northern region of the Baja California peninsula, a northwestern region of México (30°52′ N, 114°42′ W). The climate is classified as BWh (hot desert climate) according to Köppen–Geiger [20]. During summer, the ambient temperature (AT) often exceeds 45 °C and relative humidity (RH) ranges from 20 to 50%. Consequently, the climate is arid, hot, and dry with annual average rainfall of 85 mm and solar radiation reaching 1700 WS [3]. The heifer farm raised about 2000 animals annually from two dairy herds who sent them from 4 to 6 months of age until they were sent back about 2 months before the calving. The study was divided into two seasons: 3 August to 3 September 2020 was considered summer and winter was 5 February to 8 March 2021.

### 2.2. Animals, Diets, and Pens

A total of 60 non-pregnant Holstein heifers (body weight [BW] = 381 ± 5.3 kg, body condition score = 3.5 ± 1.0 units, and age = 13 ± 0.8 months), 28 in summer 2020 and 32 in winter 2021, were selected to be physiologically monitored; monitoring occurred ~1 month before 1st service in both cases. During both seasons, heifers were placed in the same pen (29.4 × 33.4 m) and provided with artificial shade made of galvanized sheet metal, feed bunks, waterers, and head locks to facilitate data collection. The pen did not have a summer cooling system. Heifers were fed a total mixed ration composed of 52% of corn silage, alfalfa hay 13%, wheat straw 10%, mixed alfalfa hay 10% (with other forages), rolled wheat grain 10%, distillers grain 3%, and 2% of mineral premix, on a dry weight basis containing 18.9% crude protein, 7.3% ash, and 15.4% neutral detergent fiber, also on a dry weight basis. Heifers were examined daily for expression of clinical signs of disease. No heifer showed such signs during the study.

### 2.3. Coat Color Classification

Heifer’s coat color was classified into two categories: (1) Black (~75% black, ~25% white), and (2) White (~75% white, ~25% black). This classification was defined by visual observation and confirmed by reviewing photo images of each heifer. During summer, 13 heifers were classified as black and 15 as white, while during winter, there were 18 black and 14 white heifers.

### 2.4. Climatic Variables

Climatic data were recorded hourly and included AT and RH, obtained from a weather station (Davis, Vantage Pro 2, Hayward, CA, USA) located at the heifer ranch. This climatic information was used to calculate the temperature-humidity index (THI) according to Hahn [21]:

THI = (0.81 × AT) + (RH/100) × (AT − 14.4) + 46.4

Climatic information is shown as overall and daily averages for each season.

### 2.5. Thermoregulatory Responses

All sampling procedures occurred during the morning (06:00 to 07:00 h) and afternoon (15:00 to 16:00 h) every third day of each season (i.e., summer = 3 August to 3 September 2020, and winter = 5 February to 8 March 2021). Respiration frequency (RF) was measured on each heifer by counting the number of breaths for 30 s. Body surface temperature (BST) was obtained using an infrared thermographic camera (Fluke Ti400, Everett, WA, USA). Two images were taken of each heifer at a distance of ~2 m; one from the front including head, nose, and eyes, and another from the right side of the body. Temperatures were recorded for eye, nose, front head, head, neck, ear, shoulder, right flank, belly, leg, loin, rump, and body. To avoid bias, heifers were photographed under shade and were free from body mud and water. The emissivity coefficient used to obtain images was 0.98. The BST’s were obtained from each image pointing at all anatomical sites listed above.

### 2.6. Hematological Profile

On days 1, 15, and 30 of each seasonal period, two blood samples were obtained from each heifer’s coccygeal vein with the 1st sample collected into 6 mL purple-capped tubes containing k3 EDTA and then transported (~10 min) to the Animal Physiology Laboratory of the ICA-UABC for direct analysis of the hematological profile in an automated liquid phase hematology equipment (MINDRAY, BC-2800 Vet, Shenzhen, China). This profile included red (RBC) and white (WBC) blood cell count, hemoglobin (HGB), hematocrit (HCT), mean corpuscular volume (MCV), mean corpuscular hemoglobin (MCH), mean corpuscular hemoglobin concentration (MCHC), erythrocyte distribution width (RDW), platelecrit (PCT), platelet count (PLT), mean platelet volume (MPV), and platelet distribution width (PDW).

### 2.7. Electrolytes, Metabolites, and Hormones

A 2nd blood sample was taken to 10 mL tubes with a red cap and clot activator, which were transported in a container with ice to the same laboratory where they were centrifuged at 3500× *g* for 15 min at 10 °C to separate serum, which was placed in duplicate 2 mL vials and stored at −20 °C for subsequent analysis of hormones, metabolites, and electrolytes. Thyroid hormones (i.e., T_3_, T_4_; Total Triiodothyronine AccuBind ELISA kit, and Total Thyroxine AccuBind ELISA kit, Monobind Inc., Lake Forest, CA, USA), progesterone (P_4_; Progesterone AccuBind ELISA kit, Monobind Inc., Lake Forest, CA, USA), and cortisol (CORT; Cortisol AccuBind ELISA kit, Monobind Inc., Lake Forest, CA, USA) were determined using commercial kits in an automated Elisa test analyzer (Thunderbolt^®^ Analyzer, Gold Standard Diagnostics, CA, USA). For T_3_, the intra- and inter-assay coefficients of variation were 5.4 and 6.7%; 1.6 and 6.1% for T_4_; 3.8 and 7.5% for progesterone; and 6.4 and 7.0% for cortisol. Serum levels of metabolites (i.e., glucose [GLU], cholesterol [COL], triglycerides [TRI], urea, total protein [TP]) were determined with a blood chemistry kit (EasyVet; KrontroLab, Morelia, Mich., México), while electrolytes (i.e., Na, K, Cl) were assessed with an electrolyte analyzer (LW E60A, Landwind, Shenzhen, China).

### 2.8. Pregnancy Rate and Services per Conception

Fifteen days after the study started, heifers were frequently observed for estrus detection and all females detected in estrus were inseminated with sexed-female semen at 12 h post-detection. Reproductive management was completed by the herd manager, who performed pregnancy diagnosis by rectal palpation 45 d after AI. Non-pregnant heifers at their first service were subjected to estrus induction protocols and AI with sexed semen for up to three services. Same reproductive management was conducted in both seasons. Pregnancy rates at 1st, 2nd, 3rd, and overall services, as well as services per pregnancy, were collected from DairyComp 305 software (Valley Agricultural Service, Los Angeles, CA, USA), which stored the complete records of each heifer.

### 2.9. Statistical Analyses

The climatic (i.e., AT, RH and THI), physiological (i.e., RF, BST), hematological (i.e., RBC, WBC, HGB, HCT, MCV, MCH, MCHC, RDW, PCT, PLT, MPV, PDW), hormones (i.e., T_3_, T_4_, P_4_, CORT), metabolites (i.e., GLU, COL, TRI, PT), and electrolyte (i.e., Na, K, Cl) measures were considered dependent variables and checked for normal distribution using the Shapiro–Wilk test using the PROC UNIVARIATE from SAS software (version 9.4, SAS Institute Inc., Cary, NC, USA). Descriptive statistics were estimated for the climatic variables, and a Student’s *t*-test was used to compare winter versus summer. Repeated measures analysis of variance was performed to determine effects of seasonal heat stress (i.e., winter and summer), coat color (i.e., black and white heifers), sampling day (i.e., 3 days), and their interactions, on the physiological, hematological, metabolites, electrolytes, and hormonal variables using PROC MIXED from SAS software. To select the best model, several variance–covariance structures were tested and the one selected was according to the Akaike and Bayesian information criteria. Models for thermoregulatory variables (RF and BST) were run separately by time of the day (i.e., morning and afternoon). Pregnancy rates were expressed in percentage and analyzed with a Chi-square test; number of services per conception was analyzed using an analysis of variance. Significance was declared at *p* ≤ 0.05.

## 3. Results

### 3.1. Climatic Variables

**Figure 1:**
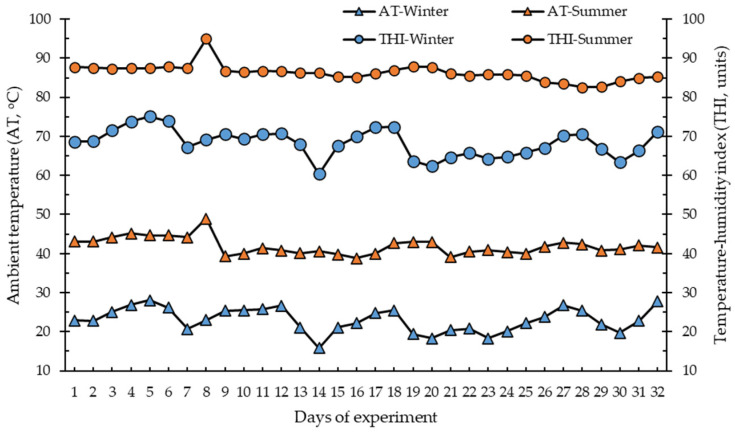
Daily averages of ambient temperature and temperature–humidity index during the winter (5 February to 8 March 2021) and summer (3 August to 3 September 2020) experimental periods.

Figure 1 and Table 1 show descriptive statistics for climatic variables by season. The average AT during summer was more than double that during winter (39 vs. 15 °C), reaching maximum AT of 49 and 28 °C in summer and winter, respectively. Average THI was 83 units in summer and 57 units in winter, with maximum THI values of 99 and 75 units, respectively. During sampling times, summer mornings had an AT average of ~27 °C and winter mornings of 8.5 °C. However, summer afternoons reached 40 °C and winter afternoons reached ~22 °C.

### 3.2. Thermoregulatory Responses

Averages and standard errors of BST and RF by season and hair coat color are in Table 2 and Table 3. During the morning, there was an interaction (*p* ≤ 0.04) of season × hair coat color for all BST variables, except (*p* ≥ 0.06) for ear, belly, leg, and eye. In the afternoon, this interaction affected (*p* ≤ 0.04) only head and right flank temperature. During mornings of winter but not summer, temperatures of nose, shoulder, loin, rib, rump, and body were higher (*p* < 0.05) in black heifers than in those white; but foot temperature was higher (*p* < 0.05) in summer white heifers and winter black heifers than in their respective counterparts. In both morning and afternoon, the head temperature was higher (*p* < 0.05) in black heifers than in white heifers in both seasons, while the right flank temperature varied (*p* < 0.05) only in winter, being higher in black heifers.

Based on main factors, temperatures of ear, belly, leg, and eye were higher (*p* < 0.05) in summer, as well as in black heifers, during the morning. Note that ear temperature was 15.2 °C higher (*p* < 0.01) in summer (33 vs. 17.8 °C, respectively), while black heifers had 0.75 °C higher (*p* < 0.05) ear temperature (25.8 vs. 25.0 °C, respectively). In addition, the BST of eye, nose, shoulder, belly, leg, foot, rump, and body during the afternoon were between 9.3 and 12 °C higher (*p* < 0.01) in summer, reaching 40 °C for all BST during summer, except for foot. However, the influence of coat color was evident as black-color heifers had temperatures between 0.83 and 1.5 °C higher (*p* < 0.01) in the eye, ear, shoulder, belly, leg, and rump during the afternoon.

In the mornings, heifers had ~12 bpm (*p* < 0.01) more in summer than in winter (48.7 vs. 36.9 bpm), but in the afternoon, this difference in RF increased (*p* < 0.01) to ~34 bpm (81.5 vs. 47.9 bpm). Hair coat color did not affect (*p* ≥ 0.10) RF at any time of day.

### 3.3. Electrolytes, Metabolites, and Hormones

Serum analyte concentration did not change (*p* ≥ 0.28) by the interaction between season and hair coat color (Table 4). Overall, heifers in winter had higher (*p* ≤ 0.03) serum concentrations of sodium, glucose, triglycerides, cholesterol, urea, and P_4_ than in summer. In contrast, serum cortisol concentration was ~40% higher (*p* < 0.01) during summer, while T_3_ and T_4_ concentrations were lower (*p* < 0.01) in this same season by ~38 and 20%, respectively. For its part, hair coat color affected only cholesterol concentration (*p* = 0.04) of all analytes, being higher in black heifers.

### 3.4. Hematological Profile

The interaction of season × hair coat color only affected (*p* < 0.01) the RDW percentage, which was higher in black than in white heifers (19.1 versus 18.9%) during winter without differences in summer (Table 5). Moreover, HGB concentration and PDW were higher (*p* < 0.01) during winter, while mean values of HTC, MCV, MCH, MCHC, and PLT were higher (*p* < 0.01) during summer. Hair coat color did not affect (*p* ≥ 0.13) the hematological parameters.

### 3.5. Pregnancy Rate and Services per Conception

Neither season nor hair color affected (*p* ≥ 0.11) the pregnancy rate at 1st, 2nd, and 3rd service, or overall. Likewise, number of services per conception was also similar (*p* ≥ 0.49) between seasons and between coat color of the heifers (Table 6).

## 4. Discussion

### 4.1. Climatic Variables

While minimum AT during summer was ~25 °C, which is the critical maximum AT for lactating dairy cows [12], according to Hahn [16], a dairy heifer with a daily gain of around 800 g has a thermoneutral zone of 0 to 15 °C, which is the AT range in which an animal reaches maximum productivity at minimal physiological cost. There was a marked difference in AT by season, which clearly indicates seasonal HS conditions for dairy heifers during summer. These conditions commonly occur in a hot desert climate. The lack of moisture in the air may lead to a more intense feeling of heat, creating a challenging environment [2]. Nutritional management and environmental physical modifications are two important practices to mitigate negative effects of HS [6,9]. As the corrals were shaded all year, heifers had access to this shelter at any time they desired, but seasonal differences were evident during the daytime. According to the THI values [9,21], winter was a season without HS conditions, with an average AT and THI of 14 °C and 50 units, respectively, values considered within the dairy heifer thermoneutral zone. In contrast, summer had an average AT and THI of 33 °C and 72 units, respectively, both considered to be HS conditions for dairy heifers.

### 4.2. Thermoregulatory Responses

The morning RF was higher in summer by 32%, a difference that increased to 67% during the afternoon (81 versus 48 bpm). Thermoregulation in heifers is a neuroendocrine mechanism that communicates information from the external environment to efferent autonomic pathways (i.e., vasoconstriction, panting, piloerection), allowing the animal to sustain a steady internal environment relative in response to a variable external environment [4,10]. In response to a high heat load with inefficient heat dissipation through sweating, heifers adjust their RF to increase evaporative cooling and decrease accumulated body heat [10]. Using multinomial logistic models, Tresoldi et al. [8] estimated the probability of different respiratory rates through various ambient temperatures for dairy heifers: when AT was between 10 and 20 °C (the thermoneutral zone), heifers had the highest probability of an RF between 30 and 50 bpm, and between 25 and 40 °C of AT above the thermoneutral zone, they had the highest likelihood to be between 50 and 70 bpm. Thus, the RF morning values of both seasons, as well as the afternoon during winter, are consistent with those from the logistic models, but RF values during the summer afternoon were higher than predicted. Thus, prior acclimatization to hot weather can be effective in young animals by allowing thermoregulation processes to counteract negative effects of HS.

In this sense, the heat load from the environment, as well as from the metabolic processes, was effectively dissipated by evaporative cooling. Moreover, the climatic conditions of deserts promote more effective evaporative cooling from skin, suggesting that the increased RF during thermal stress of our heifers reflected this adaptive mechanism. In the morning, BST of nose, shoulder, loin, rib, right flank, rump, foot, and body were higher in black coat heifers during winter, while in the summer morning, the BST of the body was higher in black heifers. Only head temperature was consistently higher in black heifers during the morning and afternoon of summer and winter. The increased temperature of several anatomic regions in black heifers supports reports that cattle with a dark coat have reduced ability to maintain thermal balance in environments with high AT and solar radiation [15]. The color of the hair coat, as well as hair numbers and density, are considered factors that impact thermoregulation when animals are exposed outdoors in geographical areas with hot weather [17,18].

Although most of the BST were within—or close to—the normal range, differences between seasons occurred. Since physiological variables were recorded during the morning and afternoon, our heifers were seemingly able to cope with severe thermal stress by activating biological cooling mechanisms (i.e., increasing RF and altering BST) to regulate their body temperature. Hair color influences the thermoregulation of ruminants, as black-coated bovines absorb ~90% of the solar radiation, while those with a white coat absorb only about 40% [22]. The estimated BSTs during the morning were below the body temperature of heifers (i.e., from 38.4 to 38.9 °C), which indicates that they were not in HS. In summer, temperatures in almost all body regions were higher in the afternoon by ~10 °C compared with morning. This point is important, especially regarding the temperature of eye and head, because they are near the brain, which provides the best indication of body core temperature [6]. As Hansen [23] found that a body surface temperature > 36 °C suggests HS in cattle, our RF, and thermography results of the heifers were collected under HS conditions during the summer afternoon. In contrast, during the summer mornings and all day in winter, they were thermoneutral. Undoubtedly, there is limited information available on the relationship between body core temperature and body surface temperature according to hair coat color in dairy heifers.

### 4.3. Blood Biochemical Parameters

In order to maintain animals in good health and production, optimum concentrations of biochemical parameters are desirable. It has been recognized that cattle responses to environmental stress among seasons have substantial effects on several serum biochemical parameters [16,24]. For instance, alterations in the hematological profile may be useful in predicting potential tolerance of cattle to hot environmental conditions [25]. During severe HS, heifers lose fluids through sweating and drooling, leading to elevated mineral concentrations, particularly sodium and potassium, which can cause metabolic acidosis [8]. It has been documented that HS leads to peripheral vasodilation and redistribution of cardiac output, which are related to increased blood volume and hemodilution [25]. The higher concentration of HTC in summer is likely due to the high AT during this season, as hot weather causes sweating—a form of water loss—and this causes hemoconcentration, which increases total erythrocyte counts and HTC [26]. In contrast, there were reductions in HGB and PDW during summer, possibly because total iron-binding capacity was higher during the cold season. Even though hemoglobin concentration was lower in summer, red blood cell indices MCV, MCH, and MCHC were higher. These indices are associated with size, shape, and quality of the red blood cells, and this change in erythrocyte morphology may be an adaptive mechanism to enhance the oxygen-carrying capacity of blood [24,25].

A reduction in hemoglobin is also related to high AT, which increases RF, as well as oxygen intake and the partial pressure of oxygen in blood. This mechanism affects erythropoiesis and can reduce hemoglobin levels [26]. While PLTs increased during summer, their distribution width decreased, suggesting that while the number of platelets increased during summer, their index of size variation decreased. However, heat-stressed heifers with elevated body temperature have been associated with decreased PLT, likely due to hemodilution during HS [27]. Under HS, plasma fluid supports an efficient thermoregulatory vehicle to reduce hyperthermia, but with negative consequences for the immune system and alterations in erythrocytic variables [28].

Heat stress lowered the metabolite concentrations glucose, triglycerides, cholesterol, and urea. Glucose is an essential fuel for energy metabolism in all animal cell types [12]. In dairy heifers, dietary carbohydrates are the main source of energy for maintenance, growth and reproduction. However, chronic hyperthermia causes prolonged negative effects on the appetite center of the hypothalamus, leading to reduced feed intake [11]. In dairy heifers, the intensity and duration of HS together affect body glucose concentration.

### 4.4. Pregnancy Rate and Services per Conception

Heat stress has been recognized as an environmental factor that impairs the reproductive performance of dairy cattle, leading to reduced pregnancy rates and, consequently, decreased fertility [29]. In arid regions, summer HS promotes the activation of the hypothalamic–pituitary–adrenal (HPA) axis to release cortisol, which, in turn, suppresses the functioning of the reproductive axis, causing a reduction in follicular growth and dominance, estrogen synthesis, and luteinizing hormone release in Holstein heifers [30]. These alterations in the reproductive hormones can lead to short estrus expression, anovulation in some cases, production of low-quality oocytes, development of small corpora lutea with limited P_4_ synthesis capacity, early embryonic losses, and, as a result, a reduced pregnancy rate [31,32]. Furthermore, it has been suggested that ovarian and hormonal failures associated with HS could be more pronounced in black-coated dairy cattle than in white-coated ones, as the first tend to have a higher body heat load and are more susceptible to hyperthermia [14]. Consequently, heifers with black coats could have more problems getting pregnant than heifers with white coats. Even so, Holstein heifers in the present study showed no detrimental effect on pregnancy rate and service per conception due to high summer AT or by having black hair coat.

It should be noted that the findings regarding the effect of hair coat color on the evaluated reproductive variables are consistent with previous reports published for Holstein cows [18,19]. However, the effect of the season on these variables was unexpected, given that seasonal HS can decrease the pregnancy rate by up to 20–30% and the confirmed conception rate by up to 40% [32]. On the same heifer ranch as our study, Correa-Calderón et al. [33] compared the reproductive performance of Holstein heifers during summer under a cooling system versus a group during the winter season, using sexed (female) semen. They found lower pregnancy rate at first service in summer than in winter (65 vs. 38%). In Iran, Joezy-Shekalgorabi et al. [34] found that heifers raised in hot semiarid regions had lower pregnancy rate and greater number of services per conception after AI with conventional or sex-sorted semen than heifers raised in temperate and cold semiarid ecosystems.

Perhaps the expected negative effects of high summer AT on pregnancy rates and services per conception did not occur because our heifers developed thermotolerance and adaptation to HS as they were born and raised in this climate, like their maternal ancestors did. This genetic improvement is advantageous for reducing the suppressive effect of the HPA axis on the reproductive axis, thereby benefiting ovarian activity and follicular estrogenic capacity [31]. Other possible reasons could be that early embryonic losses in summer were counteracted by the use of sexed semen and the improved luteal activity observed in heifers (higher P_4_ production; summer = 8.1 ng/mL vs. winter = 6.0 ng/mL). An increase in the pregnancy rate has been reported in Holstein heifers inseminated with sexed semen for females under HS because this type of semen stimulates the synthesis of embryonic cytoprotective molecules, which reduces HS damage during the embryonic development [33,35]. Moreover, sex (female)-sorted semen has shown higher capacity to penetrate cervical mucus than non-sorted semen, thus improving the pregnancy rate in cattle [36]. On the other hand, it has been widely documented that P_4_ plays an important role in maintaining a suitable uterine environment for proper maternal recognition, implantation, placental development, and consequently, embryonic development in dairy cattle [31]. Although the effect is inconsistent, the presence of high HS has been associated with reduced P_4_ concentrations in Holstein heifers and a lower pregnancy rate due to increased early embryonic losses [30,37]. However, in this study, as has also been reported in heifers from the same study site [33], high summer AT increased P_4_ concentrations, thereby improving uterine conditions for maintaining pregnancy. Therefore, summer heifers achieved pregnancy rates similar to those of winter heifers due likely to enhanced luteal activity.

## 5. Conclusions

Holstein heifers are often considered resilient due to their ability to adapt to environmental temperatures. Our results, in general, confirm this by demonstrating that black-coated heifers maintain homeothermy with minimal thermoregulatory effort under shade conditions during summer and winter in a hot and dry desert environment. During summer, Holstein heifers appeared to adjust their physiology to maintain reproductive parameters similar to those in winter. Further studies are needed to confirm an association between these variables, particularly in arid zones with high solar radiation.

## Figures and Tables

**Table 1:** Minimum (Min), maximum (Max), and average (Ave) climatic conditions during the summer and winter seasons at the experimental site.

Season	Ambient Temperature (◦C)			Relative Humidity (%)			THI (Units)		
	Min	Max	Ave	Min	Max	Ave	Min	Max	Ave
Summer	25.7	49.0	39.2	3.0	81.8	35.3	71.1	99.3	82.6
Winter	2.9	28.1	15.5	14.8	100.0	67.5	36.7	75.1	58.7

THI = Temperature–humidity index.

**Table 2:** Effect of the season × hair coat interaction on surface body temperatures of Holstein heifers under arid environmental conditions.

Items	Summer		Winter	
	White	Black	White	Black
Morning temperatures (°C)				
Nose	32.1 ± 0.44 a	32.3 ± 0.44 a	17.3 ± 0.39 b	19.2 ± 0.39 c
Head	32.7 ± 0.29 a	33.5 ± 0.29 b	18.1 ± 0.25 c	20.1 ± 0.25 d
Shoulder	34.5 ± 0.23 a	34.9 ± 0.23 a	19.2 ± 0.21 b	21.4 ± 0.21 c
Loin	33.9 ± 0.28 a	34.4 ± 0.28 a	17.0 ± 0.25 b	19.0 ± 0.25 c
Rib	34.7 ± 0.28 a	35.2 ± 0.28 a	19.5 ± 0.25 b	21.6 ± 0.25 c
Right flank	34.4 ± 0.29 a	35.1 ± 0.29 a	18.7 ± 0.26 b	20.9 ± 0.26 c
Rump	33.7 ± 0.29 a	34.3 ± 0.28 a	16.2 ± 0.25 b	17.9 ± 0.25 c
Foot	32.7 ± 0.32 a	32.0 ± 0.32 b	11.5 ± 0.28 c	12.4 ± 0.28 d
Body	34.4 ± 0.25 a	34.8 ± 0.25 a	18.3 ± 0.22 b	20.2 ± 0.22 c
Afternoon temperatures (°C)				
Head	40.0 ± 0.28 a	40.8 ± 0.28 b	28.8 ± 0.22 c	30.6 ± 0.22 d
Right flank	40.3 ± 0.36 a	41.2 ± 0.36 a	29.9 ± 0.28 b	32.5 ± 0.28 c

a, b, c, d Averages with different letters indicate differences at *p* ˂ 0.01.

**Table 3:** Morning and afternoon body surface temperatures during the summer and winter seasons in white and black Holstein heifers under arid environmental conditions.

Items	Season (S)			Hair Color (C)			*p*-Values		
	Summer	Winter	SEM	White	Black	SEM	S	C	S × C
*Morning temperatures (°C)*									
Head	33.1	19.1	0.19	25.4	26.8	0.19	0.01	0.01	0.03
Nose	32.2	18.3	0.30	24.7	25.7	0.29	0.01	0.01	0.04
Ear	33.2	18.0	0.26	25.3	26.0	0.25	0.01	0.01	0.16
Eye	33.9	20.9	0.25	27.0	27.8	0.24	0.01	0.01	0.06
Shoulder	34.7	20.3	0.15	26.8	28.2	0.15	0.01	0.01	<0.01
Loin	34.1	18.0	0.19	25.4	26.7	0.19	0.01	0.01	<0.01
Rib	34.9	20.5	0.18	27.1	28.4	0.18	0.01	0.01	<0.01
Belly	34.4	16.8	0.19	25.2	26.0	0.19	0.01	0.01	0.07
Right flank	34.7	19.8	0.19	26.5	28.0	0.19	0.01	0.01	<0.01
Leg	34.5	17.2	0.19	25.6	26.1	0.19	0.01	0.01	0.16
Foot	32.4	22.0	0.21	22.1	22.2	0.21	0.01	0.01	<0.01
Rump	34.0	17.1	0.19	25.0	26.1	0.19	0.01	0.01	0.04
Body	34.6	19.3	0.16	26.4	27.5	0.16	0.01	0.01	<0.01
*Afternoon temperatures (°C)*									
Head	40.4	29.7	0.18	34.4	35.7	0.17	0.01	0.01	0.04
Nose	38.8	28.6	0.23	33.6	33.8	0.23	0.01	0.01	0.53
Ear	40.7	29.4	0.18	34.6	35.5	0.19	0.01	0.01	0.21
Eye	40.3	30.3	0.19	34.8	35.9	0.19	0.01	0.01	0.16
Shoulder	40.4	31.1	0.18	35.3	36.3	0.20	0.01	0.01	0.14
Loin	41.1	30.2	0.22	34.9	36.4	0.22	0.01	0.01	0.06
Rib	40.2	31.7	0.23	35.2	36.7	0.22	0.01	0.01	0.10
Belly	40.7	29.5	0.23	34.6	35.7	0.23	0.01	0.01	0.60
Right flank	40.7	31.2	0.22	35.1	36.8	0.22	0.01	0.01	0.01
Leg	41.3	29.6	0.22	34.8	36.1	0.21	0.01	0.01	0.73
Foot	39.4	26.2	0.40	32.9	32.7	0.39	0.01	0.01	0.68
Rump	41.5	29.7	0.20	35.0	36.2	0.19	0.01	0.01	0.24
Body	40.7	30.6	0.22	35.1	36.3	0.21	0.01	0.01	0.42
*Respiratory frequency* (Breaths per minute)									
Morning	48.70	36.90	0.41	43.2	42.3	0.42	<0.01	0.10	0.37
Afternoon	80.90	48.40	0.50	64.6	64.7	0.48	<0.01	0.91	0.63

SEM = Standard error of the mean.

**Table 4:** Serum analyte concentrations during summer and winter seasons in white and black Holstein heifers under arid environmental conditions.

Items	Season (S)			Hair Color (C)			*p*-Values		
	Summer	Winter	SEM	White	Black	SEM	S	C	S × C
*Electrolytes (mmol/L)*									
Sodium	129.4	130.2	0.27	129.6	129.9	0.26	0.03	0.40	0.49
Potassium	4.62	4.53	0.06	4.59	4.56	0.06	0.33	0.74	0.72
Chloride	104.1	103.9	0.21	104.1	104.0	0.20	0.43	0.71	0.99
*Metabolites (mg/dL)*									
Glucose	86.7	100.7	0.94	93.6	93.9	0.95	<0.01	0.83	0.49
Triglycerides	23.8	35.0	0.86	29.5	30.2	0.87	<0.01	0.56	0.92
Cholesterol	95.5	109.5	1.62	100.2	104.8	1.62	<0.01	0.04	0.97
Total protein	7.09	7.25	0.07	7.23	7.10	0.07	0.13	0.20	0.34
Urea	23.3	25.3	0.48	24.1	24.4	0.47	<0.01	0.68	0.65
*Hormones*									
Progesterone (ng/mL)	8.80	6.00	0.57	7.50	7.30	0.56	<0.01	0.87	0.28
Triiodothyronine (ng/mL)	0.96	1.55	0.05	1.28	1.22	0.05	<0.01	0.38	0.33
Thyroxine (µg/dL)	4.80	5.90	0.13	5.50	5.20	0.13	<0.01	0.08	0.95
Cortisol (µg/dL)	5.20	2.80	0.35	4.30	3.80	0.35	<0.01	0.17	0.92

SEM = Standard error of the mean.

**Table 5:** Hematology profile during summer and winter seasons in white and black Holstein heifers under arid environmental conditions.

Items	Season (S)			Hair Color (C)			*p*-Values		
	Summer	Winter	SEM	White	Black	SEM	S	C	S × C
White blood cells (×10^12^/L)	10.4	10.8	0.57	11.0	10.2	0.56	0.58	0.13	0.39
Red blood cells (×10^9^/L)	8.33	8.26	0.17	8.26	8.32	0.16	0.67	0.71	0.26
Hemoglobin (g/L)	10.4	11.7	0.16	11.0	11.0	0.15	<0.01	0.99	0.36
Hematocrit (%)	33.8	31.3	0.52	32.2	32.9	0.49	<0.01	0.31	0.17
Mean corpuscular volume (fL)	41.6	38.8	0.41	40.2	40.4	0.40	<0.01	0.93	0.71
Mean corpuscular hemoglobin (pg)	14.2	12.8	0.14	13.6	13.5	0.13	<0.01	0.95	0.79
MCHC (g/L)	34.6	33.0	0.14	33.9	33.7	0.14	<0.01	0.22	0.34
Erythrocyte distribution width (%)	19.0	18.6	0.32	18.8	18.7	0.31	0.12	0.91	<0.01
Platelet count (×10^9^/L)	348.9	268.9	18.8	323.1	294.8	16.7	<0.01	0.23	0.44
Mean platelet volume (fL)	5.00	5.00	0.04	5.00	5.00	0.04	0.51	0.14	0.48
Platelet distribution width (%)	15.8	16.0	0.05	15.9	15.9	0.05	<0.01	0.95	0.97
Platelecrit (%)	0.17	0.14	0.01	0.15	0.16	0.01	0.11	0.93	0.28

MCHC = Mean corpuscular hemoglobin concentration; SEM = Standard error of the mean.

**Table 6:** Pregnancy rates and services per conception during summer and winter seasons in white and black Holstein heifers under arid environmental conditions.

Items	Season (S)		Hair Color (C)		*p*		
	Summer	Winter	White	Black	S	C	S × C
Pregnancy rate (%)							
1st service	53.6 (15/28)	56.3 (18/32)	48.3 (14/29)	61.3 (19/31)	0.84	0.47	0.13
2nd service	46.2 (6/13)	42.9 (6/14)	53.3 (8/15)	33.3 (4/12)	0.97	0.11	0.35
3rd service	100 (7/7)	87.5 (7/8)	71.4 (5/7)	100 (8/8)	0.30	0.44	0.70
Overall	100 (28/28)	96.9 (31/32)	93.1 (27/29)	100 (31/31)	0.68	0.84	0.89
Services/conception (n)	1.79 ± 0.19	1.67 ± 0.21	1.77 ± 0.22	1.69 ± 0.19	0.66	0.77	0.49

## Data Availability

Data from this study is available upon a rational request.
